# Elevated level of circulating calprotectin correlates with severity and high mortality in patients with COVID‐19

**DOI:** 10.1002/iid3.1212

**Published:** 2024-03-13

**Authors:** Haoran Zhang, Qingyu Zhang, Kun Liu, Zenong Yuan, Xiqiang Xu, Jun Dong

**Affiliations:** ^1^ Department of Orthopaedics Shandong Provincial Hospital Affiliated to Shandong First Medical University Jinan Shandong Province China; ^2^ Graduate School of Education Shandong Sport University Jinan Shandong Province China

**Keywords:** biomarker, calprotectin, COVID‐19, meta‐analysis, systematic review

## Abstract

**Background:**

Patients with coronavirus disease‐2019 (COVID‐19) are characterized by hyperinflammation. Calprotectin (S100A8/S100A9) is a calcium‐ and zinc‐binding protein mainly secreted by neutrophilic granulocytes or macrophages and has been suggested to be correlated with the severity and prognosis of COVID‐19.

**Aim:**

To thoroughly evaluate the diagnostic and prognostic utility of calprotectin in patients with COVID‐19 by analyzing relevant studies.

**Methods:**

PubMed, Web of Science, and Cochrane Library were comprehensively searched from inception to August 1, 2023 to retrieve studies about the application of calprotectin in COVID‐19. Useful data such as the level of calprotectin in different groups and the diagnostic efficacy of this biomarker for severe COVID‐19 were extracted and aggregated by using Stata 16.0 software.

**Results:**

Fifteen studies were brought into this meta‐analysis. First, the pooled standardized mean differences (SMDs) were used to estimate the differences in the levels of circulating calprotectin between patients with severe and non‐severe COVID‐19. The results showed an overall estimate of 1.84 (95% confidence interval [CI]: 1.09–2.60). Diagnostic information was extracted from 11 studies, and the pooled sensitivity and specificity of calprotectin for diagnosing severe COVID‐19 were 0.75 (95% CI: 0.64–0.84) and 0.88 (95% CI: 0.79–0.94), respectively. The AUC was 0.89 and the pooled DOR was 18.44 (95% CI: 9.07–37.51). Furthermore, there was a strong correlation between elevated levels of circulating calprotectin and a higher risk of mortality outcomes in COVID‐19 patients (odds ratio: 8.60, 95% CI: 2.17–34.12; *p* < 0.1).

**Conclusion:**

This meta‐analysis showed that calprotectin was elevated in patients with severe COVID‐19, and this atypical inflammatory cytokine might serve as a useful biomarker to distinguish the severity of COVID‐19 and predict the prognosis.

## Graphical abstract

This article showed that calprotectin may be a useful biomarker to distinguish the severity and predict the prognosis of COVID‐19.

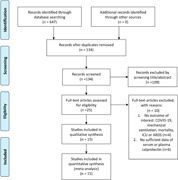

## 1 INTRODUCTION

In the last few years, more than 750 million cases of coronavirus disease‐2019 (COVID‐19) have been recorded worldwide and three billion people have been vaccinated.^1^, ^2^ Common symptoms at the onset of COVID‐19 include fever, cough without or without sputum, sore throat, nasal congestion, dizziness, shortness of breath, fatigue, myalgia, and headache.^3^ In fact, the clinical course of this disease can range from asymptomatic infection to severe and even fatal illness. It is estimated that severe cases of COVID‐19 account for about 1/5 of all cases, and the fatality rate for patients over 85 years old is as high as 30%.^4^ To date, seven million COVID‐19 deaths have been reported to WHO.^2^ As the COVID‐19 pandemic continued, various SARS‐CoV‐2 variants emerged and rapidly spread.^5^ A sequence abundance analysis revealed that the Omicron variant is the most widely distributed and transmissible strain, but the disease severity is less.^6^ Hospitalization risk, ICU admission rate, mechanical ventilation use rate, and hospitalization period of Omicron variant infection were shorter.^7^ COVID‐19 has been shown to be a highly inflammatory disease, and patients with severe symptoms are characterized by elevated serum levels of various cytokines.^8^, ^9^ Unfortunately, although many cytokines have been evaluated as markers for determining the severity or prognosis of COVID‐19, there is not a universal endorsement.

Calprotectin (S100A8/S100A9) is a calcium‐ and zinc‐bound heterodimeric molecule consisting of two heavy chains and one light chain noncovalently linked with a classical helix‐loop‐helix structural domain.^10^, ^11^ This atypical inflammatory cytokine is mainly produced by neutrophilic granulocytes or macrophages at sites of inflammation in a calcium‐dependent manner.^12^ Calprotectin could modulate cyclooxygenase activity to regulate the expression of prostaglandin analogs, promoting the adhesion of phagocytes to the vascular endothelium and thrombi formation.^13^, ^14^ Moreover, it has potent antimicrobial effects against various bacterial and fungal pathogens.^15^ As an acute phase reactant, the expression level of calprotectin is often increased following infection, trauma, and inflammatory diseases such as rheumatoid arthritis, ankylosing spondylitis, and inflammatory bowel disease, serving as a biomarker to help with the diagnosis. In particular, fecal calprotectin has become one of the most important indicators for monitoring inflammatory bowel disease.^16^

For COVID‐19, which is highly transmissible, there is still an unmet need to determine its severity and predict mortality outcomes. Some biomarkers such as interleukin‐6 (IL‐6), C‐reactive protein (CRP), d‐dimer, ferritin, and serum amyloid A (SAA) have been recognized to assess the severity of COVID‐19.^17^, ^18^ Both circulating and fecal calprotectin were reported to be associated with COVID‐19, and this article focused on the clinical significance of serum and plasma calprotectin for COVID‐19. The aim of this study is to find a biomarker that can predict sudden and unexpected clinical deterioration and disease severity of COVID‐19. Not only for early screening and diagnosis but also to predict the risk of death. A pairwise meta‐analysis was used to reduce bias due to the insufficient number of studies and to improve the accuracy of the conclusion.

## 2 MATERIALS AND METHODS

The protocol for this review is registered on PROSPERO (International prospective register of systematic reviews) and the registration number is CRD42023452343. This study was reported in line with PRISMA (Preferred Reporting Items for Systematic Reviews and Meta‐Analyses) guidelines.^19^ Electronic searches, reference lists screening, study selection, data extraction, methodological quality assessment and pooling of outcome estimates were performed by two authors (ZHR and ZQY) independently. For any ambiguities, a consensus was reached through active discussions or consulting with a third author (DJ). All required data were retrieved from published articles; thus, informed consent or ethical approval was not required.

### 2.1 Data source and search strategy

Three electronic databases including PubMed, EMBASE, and the Cochrane library (from inception to July 11, 2023) were searched by using a combination of keywords and mesh terms ([Calprotectin, S100A8/A9] AND [COVID‐19, SARS‐COV‐2, Novel coronavirus]) to obtain relevant studies. No restrictions on publication date, language, or the journal of publication were imposed. Titles and abstracts were independently screened by using the selection criteria to determine the eligible studies. Then, the full texts of the remaining records were carefully evaluated to include or exclude these studies accordingly. Negotiation and discussion were reached for any paper with controversial content to eliminate doubts. Lastly, a manual search was conducted in the reference list of related studies (especially reviews, meta‐analyses, and the included studies) for additional eligible literature.

### 2.2 Inclusion and exclusion criteria

Articles in which adults had COVID‐19 were enrolled and tested for serum or plasma calprotectin were included. These studies should delineate mild and severe, or can indirectly differentiate mild and severe based on intensive care unit (ICU) care, acute respiratory distress syndrome (ARDS), mechanical ventilation, or death. Our exclusion criteria were articles with incomplete information, non‐English language articles, and articles with the control group involved other inflammatory diseases.

### 2.3 Data extraction

Following data were extracted from qualified research: name of first author, study design, region, inclusion interval of patients, number of patients, comorbidities, outcome of interest, gender, age, death, diagnostic information of calprotectin (true positive/TP, false positive/FP, false negative/FN, and true negative/TN), mean and associated standard deviation (SD) of calprotectin, and odds ratio of mortality.

### 2.4 Quality assessment

The QUADAS‐2 tool was used to assess the quality of individual studies and the Review Manager software (version 5.3) was used to draw the graph of the quality. The QUADAS‐2 tool consisted of the following four parts: Patients Selection, Index Test, Reference Standard, and Flow and Timing. All parts were evaluated in terms of risk of bias and the former three parts were evaluated in terms of applicability concerns.

### 2.5 Approach to evidence synthesis and analysis

STATA 16.0 software was used to merge the effect sizes of the diagnostic test, calculating the combined sensitivity, specificity, positive likelihood ratio (PLR), negative likelihood ratio (NLR), diagnostic odds ratio (DOR). The range of DOR was 0 to infinity, and the larger the value, the better the diagnostic efficiency.^20^ The summary receiver operating characteristic curve (SROC) was constructed to obtain the area under the curve (AUC). The AUC of SROC ranged from 0 to 1: AUC of <0.5 indicates no diagnostic significance; AUC of 0.5–0.7, low diagnostic accuracy; AUC of 0.7–0.9, moderate diagnostic performance; and AUC of >0.9, high diagnostic accuracy.^21^ The SROC curve was observed to assess whether there is apparent shoulder‐arm effect. The Spearman correlation coefficient is calculated, and if the *p* > .05, the heterogeneity caused by the threshold effect is considered to be absent. Then we performed the Cochran *Q* test and *I*² test to estimate the existence and severity of heterogeneity. Significant heterogeneity is considered when *p* < .1 and *I*² > 50%. Deek's funnel plot was used to assess whether there was publication bias. Symmetry of the funnel plot suggested no publication bias, asymmetry of the funnel plot, and *p* < .05 of linear regression analysis suggested the existence of publication bias.

If continuous variables appear as mean and standard deviation, these data were extracted directly. If they appear as median and interquartile range, they need to be converted to mean and standard deviation before extraction according to Luo's and Wan's methods.^22^, ^23^ Due to the different methods and units for assessing the calprotectin level, the standardized mean difference was adopted as the effect size.

## 3 RESULTS

### 3.1 Search results

**Figure 1:**
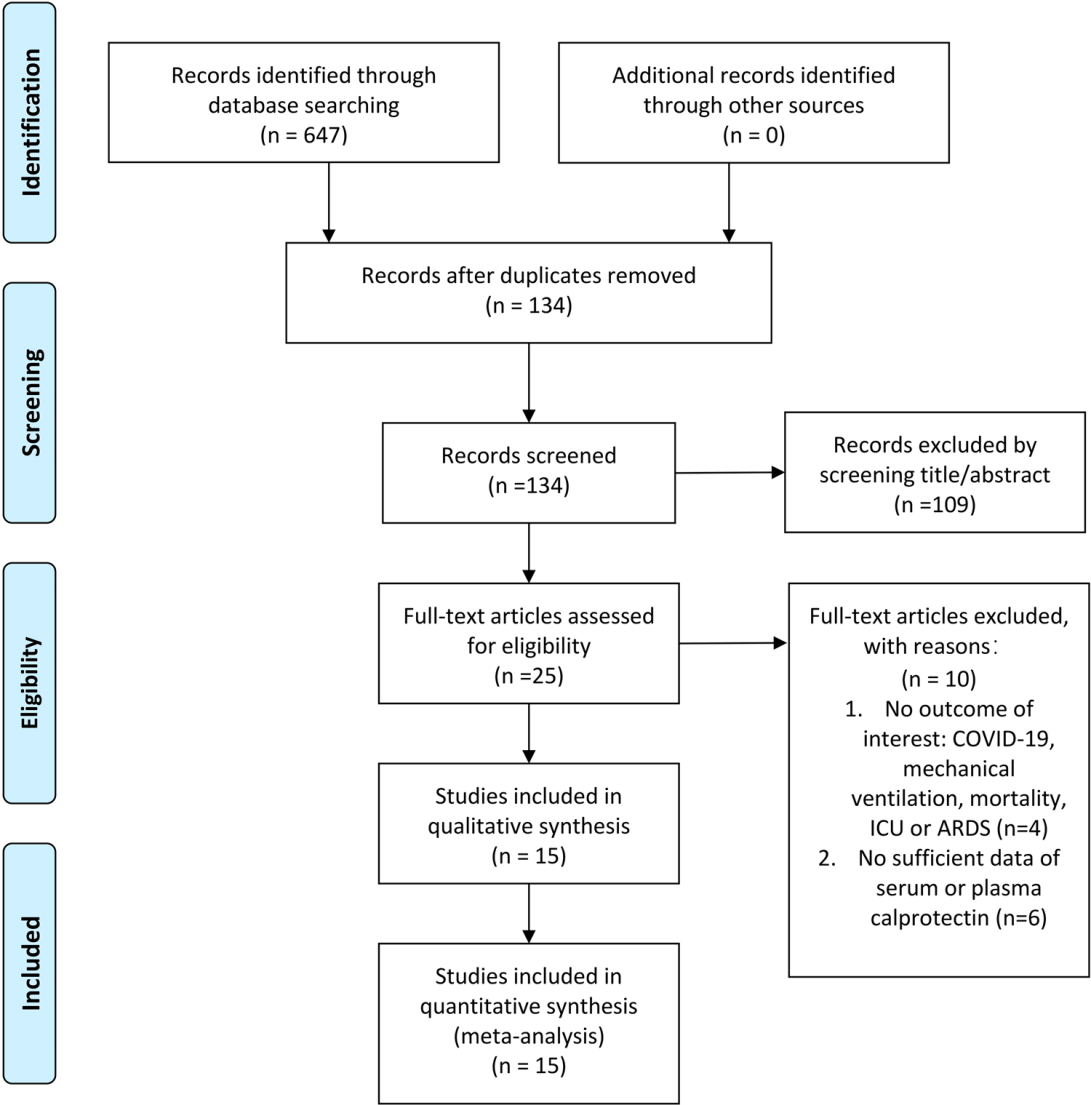
PRISMA 2009 flow diagram. A total of 647 records were retrieved, after inclusion and exclusion criteria, the final 15 studies were included.

The flow chart of the literature screening process is shown in Figure 1. A total of 647 records were identified from electronic database and 134 studies were left after eliminating duplicates. Through assessing titles/abstracts according predefined criteria, 109 records were excluded. Because of the absence of information about COVID‐19, mechanical ventilation, mortality, ICU, or ARDS, which were outcomes of our interest, four studies were further discarded. Since there was no sufficient data of serum or plasma calprotectin, six others studies were also considered ineligible for this meta‐analysis. Finally, 15 studies^24^, ^25^, ^26^, ^27^, ^28^, ^29^, ^30^, ^31^, ^32^, ^33^, ^34^, ^35^, ^36^, ^37^, ^38^ involving 2753 patients were included in the quantitative analysis. Among the 15 studies, 11^24^, ^26^, ^27^, ^28^, ^29^, ^31^, ^32^, ^33^, ^34^, ^35^, ^38^ assessed the diagnostic value of calprotectin, five^25^, ^31^, ^34^, ^35^, ^36^ reported the mortality and eight^24^, ^27^, ^29^, ^30^, ^32^, ^35^, ^36^, ^37^ presented the level of calprotectin. The methods used to detect calprotectin in all 15 studies were enzyme‐linked immunosorbent assay (ELISA), chemiluminescent immunoassay, turbidimetric assay, and quantitative reverse‐transcription polymerase chain reaction (RT‐qPCR). ELISA was used in eight papers, chemiluminescent immunoassay was used in two papers, turbidimetric assay was used in five papers and RT‐qPCR was used in one paper. And one article used both turbidimetric assays and chemiluminescent immunoassays. The baseline characteristics of enrolled patients are shown in Table 1.

**Table 1:** Baseline characteristics of included studies.

Autor	Study design	Region	Inclusion interval of patients	Inclusion number of patients	Comorbidities	Outcome of interest	Male, *n* (%)	Age, years	% Deaths	Gold standard	Mild COVID‐19 calprotectin range	Severe
Shokri‑Afra et al.^28^	Prospective study	Iran	2020.09–2020.11	COVID‐19 *n* = 76, non‐COVID *n* = 24	Diabetes, hypertension, cardiovascular disease, chronic kidney disease, chronic liver disease	Serum calprotectin	COVID‐19 *n* = 39(51.3%), non‐COVID‐19 *n* = 16 (66.7%)	COVID‐19 52.1 ± 17.2, non‐COVID‐19 52.83 ± 22.61^a^	5.30%	ELISA	NR	NR
Cardiero et al.^25^	Retrospective study	Italy	2020.03.10–2020.04.30; 2020.12.08–2021.04.30	COVID‐19 *n* = 195	Decreased lung function, diabetes, heart disease, hypertension	Circulating calprotectin	94 (48.2%)	55 (34–67)^b^	5.13%	Particle‐enhanced turbidimetric immunoassay and chemiluminescent immunoassay	NR	NR
Norman et al.^26^	NR	USA	2020 spring	COVID *n* = 157	Diabetes, heart disease, renal disease, lung disease, autoimmune, cancer, history of stroke, obesity, hypertension, history of smoking	Circulating calprotectin	92 (59%)	58 ± 17^a^	22%	Chemiluminescent immunoassay	NR	NR
Kaya et al.^27^	Retrospective cross‐sectional cohort study	Turkey	2020.11.01–2020.12.10	COVID‐19 *n* = 80	Diabetes mellitus, hypertension, coronary artery disease, renal failure, chronic respiratory disease	Circulating calprotectin	ICU *n* = 24 (63.2%); non‐ICU *n* = 19(45.2%)	ICU 70.8 ± 12.8, non‐ICU 62.5 ± 17.2^a^	ICU 57.1%	Immunoturbidimetric assay	37.9 (30.3–46.2)^b^ (ng/mL)	44.8 (34.5–61.5)^b^
Shokri‑Afra et al.^24^	Prospective study	Iran	2020.09–2020.11	COVID‐19 *n* = 70, non‐COVID 19 *n* = 19	Hypertension, Diabetes mellitus, cardiovascular disease, dyslipidemia chronic kidney disease, Chronic liver disease,	Serum calprotectin	COVID‐19 *n* = 36 (51.4); non‐COVID 19 *n* = 12 (63.2)	COVID‐19 51.47 ± 17.28, non‐COVID 19 47.74 ± 21.27^a^	COVID‐19 5.71%	ELISA	2013 ± 1074^a^ (ng/mL)	5436 ± 940^a^ (ng/mL)
Lee et al.^29^	Retrospective study	Korea	2020.07.04–2020.11.15	COVID‐19 *n* = 105	Diabetes, hypertension, cardiovascular diseases, renal disease, cancer, autoimmune disease, dementia/mental retardation, pregnancy, hyperlipidemia, hepatitis B virus infection, obesity	Serum calprotectin	45 (42.9%)	68.0 ± 18.8^a^	8.60%	ELISA	2.60 (1.40–­5.28)^b^ (μg/mL)	12.60 (8.10–­18.50)^b^
Shrivastava et al.^30^	Retrospective study	India	2020.05–2020.09	Healthy *n* = 19, mild *n* = 32, severe *n* = 31	NR	Circulating calprotectin	Healthy *n* = 10 (53%), mild *n* = 19 (59%); severe *n* = 21 (68%)	Healthy 27 (18–33), mild 40 (15‐67), severe 59 (30–85)^b^	NR	ELISA	16,589 ± 2651^a^ (ng/mL)	18,183 ± 2512^a^
Morgane Ducastle et al.^31^	Retrospective monocentric study	France	2020.04‐2020.05	Mild *n* = 31, moderate *n* = 36, severe *n* = 36, critical *n* = 57	Overweight, hypertension, diabetes, chronic kidney disease, cardiovascular disease, chronic respiratory failure, systemic autoimmune Disease	Plasma calprotectin	Mild *n* = 11 (35.5%); moderate *n* = 20 (55.6%); severe *n* = 18 (50%); critical *n* = 43 (75.4%)	Mild 47.4 (32.4–57.1), moderate 63.9 (49.1–75.8), severe 64.7 (58.5–74.9), critical 61.9 (51.2–73.9)^b^	Critical 29.8%	Turbidimetric assay	NR	NR
Shi et al.^32^	Prospective study	USA	NR	Room air *n* = 41, noninvasive supplemental oxygen *n* = 71, mechanical ventilation *n* = 60	Diabetes, ischemic heart disease, renal disease, lung disease, autoimmune, cancer, history of stroke, obesity, hypertension, immune deficiency, history of smoking	Circulating calprotectin	COVID‐19 *n* = 97 (56%)	61.48 ± 17.7^a^	NR	ELISA	3365 ± 3146^a ^(ng/mL)	8039 ± 7031^a^
Silvin et al.^33^	Cohort	France	NR	Negative control *n* = 10, mild *n* = 3, moderate *n* = 4, severe *n* = 7	Overweight, cardiovascular, diabetes, respiratory, cancer	Plasma calprotectin	All *n* = 12 (50%); negative control *n* = 6 (60%), mild *n* = 1 (33%), moderate *n* = 1 (25%), severe *n* = 4 (57%)	60 (54–68)^b^	All *n* = 1 (4%), severe *n* = 1(14%)	RT‐qPCR	NR	NR
Chen et al.^34^	Retrospective study	China	NR	ICU *n* = 40; non‐ICU *n* = 81	Hypertension, diabetes, coronary artery heart disease, cancer, COPD, immunodeficiency	Serum calprotectin	All *n* = 77 (63.6%); non‐ICU *n* = 49 (60.5%), ICU *n* = 28 (70.0%)	63 (53–70)^b^	All *n* = 36 (29.8%); non‐ICU *n* = 3 (3.7%), ICU *n* = 33 (82.5%)	ELISA	NR	NR
Bauer et al.^35^	Prospective study	Germany	NR	ICU *n* = 8, MOF within 72 h *n* = 4, total MOF *n* = 6, 90‐day mortality *n* = 2	NR	Serum calprotectin	COVID‐19 *n* = 8 (42%)	67.6 (53.9–72.0)^b^	10.50%	Turbidimetric assay	2.08 (1.36–2.59)^b^ (mg/L)	3.77 (1.90–5.16)^b^
Luis et al.^36^	Observational study	Spain	2020.03‐2020.04	Survivors *n* = 58; nonsurvivors *n* = 8	Hypertension, diabetes mellitus, non‐asthma respiratory disease, cardiovascular disease, chronic kidney disease, immunosuppression	Circulating calprotectin	43 (65.2%)	61 ± 16^a^	12.1% (8/66)	Particle‐enhanced turbidimetric immunoassay	6.0 (2.8–10.8) ^b^(mg/dL)	19.3 (10.4–30.5)^b^
Gupta et al.^37^	NR	Dubai	2020.05‐ 2020.06	Moderate *n* = 15, severe *n* = 15, control *n* = 10	NR	Plasma calprotectin	NR	Moderate 48 ± 8.1, severe 55 ± 8.3^a^	33.3% (5/15)	ELISA	0.8972 ± 0.1868^a^ (ng/mL)	3.626 ± 0.748^a^ (ng/mL)
Kassianidis et al.^38^	NR	Greece	2020.04‐2020.11	Control *n* = 40, asymptomatic *n* = 19; moderate *n* = 42, severe *n* = 78, ARDS and were on MV *n* = 42	Type 2 diabetes mellitus, chronic heart failure, Chronic renal disease, coronary heart disease, dyslipidemia, hypothyroidism, hypertension, stroke, atrial fibrillation, COPD	Serum calprotectin	Control *n* = 28(70%), asymptomatic *n* = 12(63.2%), moderate *n* = 25(59.5%), severe *n* = 56(71.8%), ARDS and were on MV *n* = 35(83.3%)	Control 58.3 ± 15.8, asymptomatic 59.5 ± 10.8, moderate 55.3 ± 14.8, severe 61.4 ± 13.9, ARDS and were on MV 64.9 ± 12.8^a^	NR	ELISA	NR	NR

Abbreviations: ARDS, acute respiratory distress syndrome; COPD, chronic obstructive pulmonary disease; COVID‐19, coronavirus disease‐2019; ELISA, enzyme‐linked immunosorbent assay; ICU, intensive care unit; MOF, multiorgan failure; MV, mechanical ventilation; NR, not reported.

^a^ Age, mean ± SD.

^b^ Age, median (first quantile‐third quantile).

### 3.2 Quality assessment

**Figure 2:**
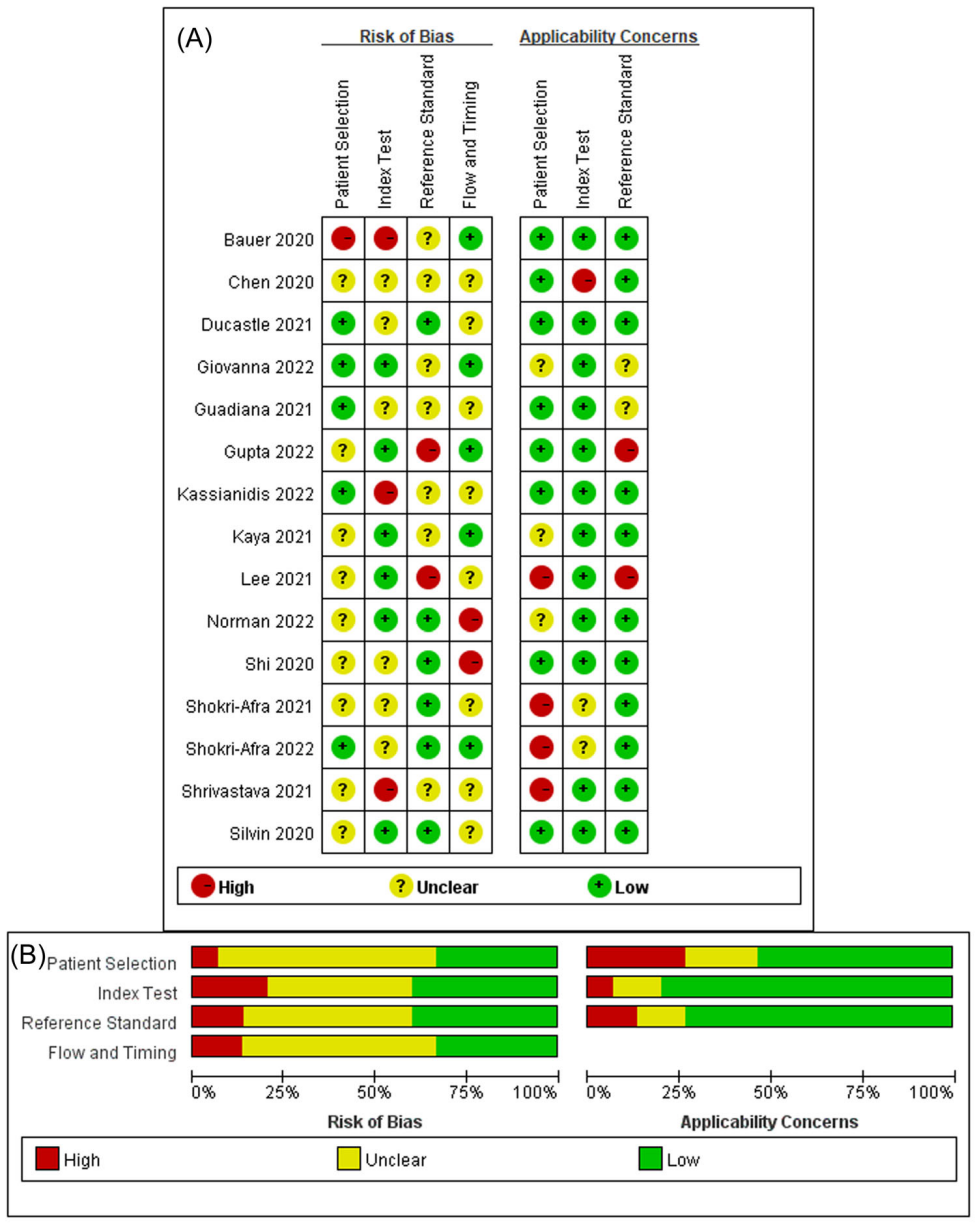
Review authors' judgements about each risk of bias item for included studies. (A) Risk of bias summary; (B) risk of bias graph presented as percentages.

The studies we included were all of high quality (Figure 2). The risk assessment of patient selection was not satisfactory, four studies did not avoid case‐control studies, and six studies did not include consecutive or random sample of patients. This is the most important cause of patient selection bias.

### 3.3 Severity of COVID‐19 and calprotectin levels

**Figure 3:**
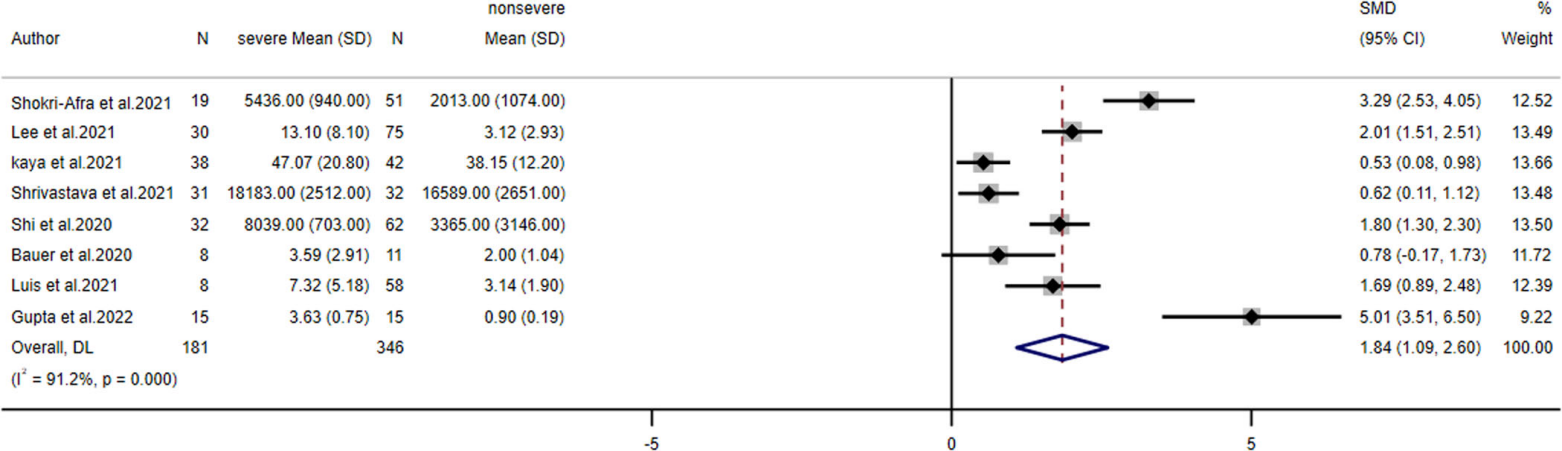
Forest plot of calprotectin levels.

On account of the *I* ^2^ = 91.2%, a random effect model was used and the pooled SMD of calprotectin between patients with severe COVID‐19 and those with nonsevere COVID‐19 was 1.84 (95% confidence interval [CI]: 1.09–2.60) (Figure 3). The symmetric distribution of Deek's funnel plot and the result of Egger's test result (*p* = .124) both indicated low publication bias (Figure SS1). Next, a sensitivity analysis of the pooled results of changes in calprotectin levels was performed by excluding the eight included studies individually (Figure SS2). The results showed no significant change in the final effect size, which demonstrated that the conclusion was robust and reliable.

### 3.4 Diagnostic performance of calprotectin in predicting COVID‐19 severity

**Figure 4:**
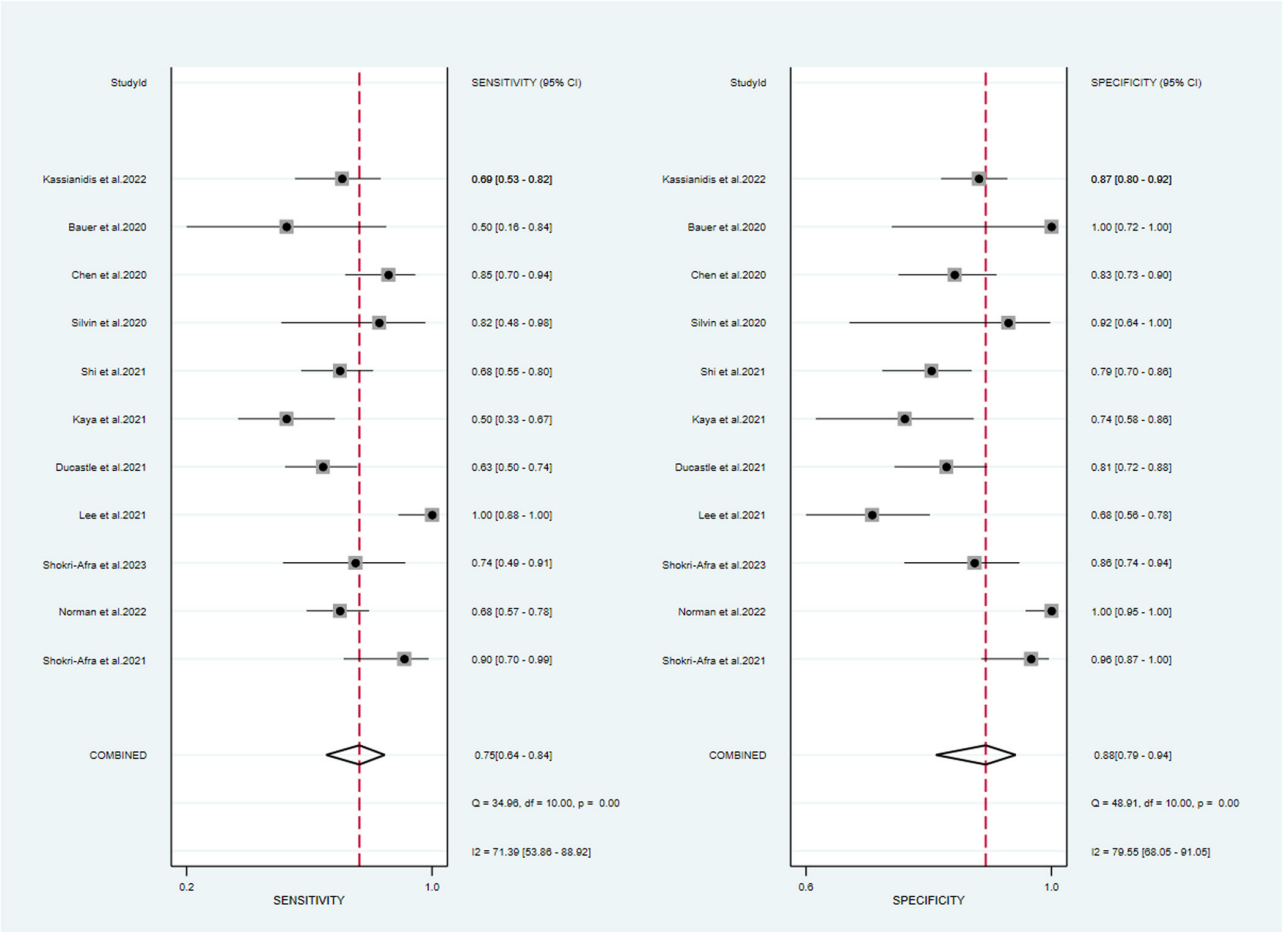
Forest plots of sensitivity and specificity of calprotectin diagnosis value for COVID‐19 severity.

**Figure 5:**
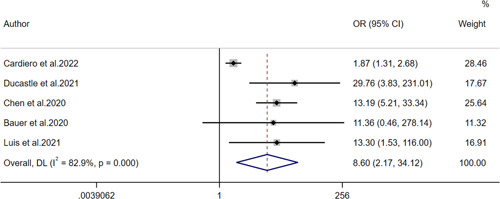
Forest plot for the combined diagnostic odds ratio of calprotectin.

**Figure 6:**
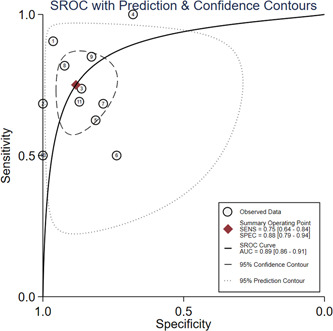
Summary receiver operating characteristic curve of COVID‐19 severity detected by calprotectin.

The pooled sensitivity, specificity, PLR, NLR, and DOR as well as the associated 95% CI were 0.75 (95% CI: 0.64–0.84), 0.88 (95% CI: 0.79–0.94), 6.4 (95% CI: 3.5–11.6), 0.28 (95% CI: 0.19–0.42), 18.44 (95% CI: 9.07–37.51), respectively (Figures 4 and 5). All the above indicators suggested that calprotectin had a satisfactory efficiency for determining the severity of COVID‐19. Furthermore, the SROC curve gave an AUC of 0.89 (95% CI: 0.86–0.91) (Figure 6). The publication bias was assessed by plotting Deek's funnel plot (Figure SS3). It is obvious that the 11 included studies were evenly distributed on both sides of the regression line. The difference was not statistically significant (*p* = .83), suggesting that the included studies had a low risk of publication bias.

### 3.5 The mortality rates

**Figure 7:**
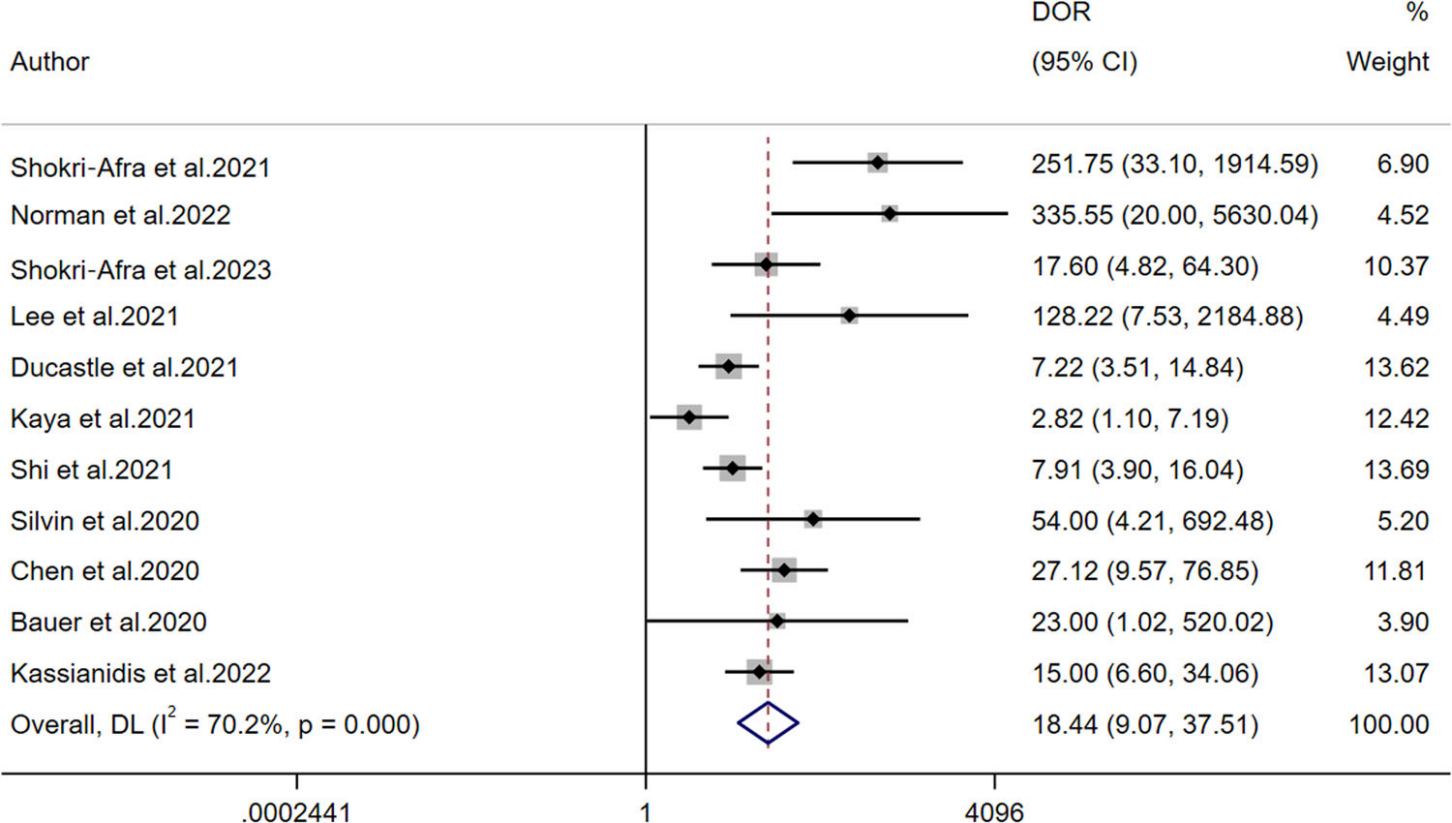
Forest plot for the odds ratio of mortality.

Five studies provided data related to mortality outcomes, and OR with 95%CI was used to evaluate mortality outcomes (Figure 7). High heterogeneity can be observed in the forest plots (*I* ^2^ = 82.9%, *p* < .001). Therefore, the random effects model was selected. The pooled OR and 95% CI were 8.60 (95% CI: 2.17–34.12), unveiling that COVID‐19 mortality is positively correlated with the calprotectin level.

## 4 DISCUSSION

After infection, neutrophil counts increased and natural killer cells decreased, proinflammatory cytokines such as tumor necrosis factor‐α (TNF‐α), IL‐1, IL‐2, IL‐6, and IFN‐γ‐inducible protein 10 (IP‐10) was also abnormally elevated.^39^, ^40^ Due to the intrinsic cytotoxic and proinflammatory properties of calprotectin, the level of this atypical cytokine is demonstrated to be associated with the development of inflammatory/autoimmune diseases (e.g., rheumatoid arthritis, systemic lupus erythematosus, and connective tissue diseases).^41^, ^42^, ^43^, ^44^ COVID‐19 is actually characterized by a cytokine storm, in which the immune system plays a regulatory role in summoning cytokines to attack pathogens.^45^ This meta‐analysis was performed to assess the diagnostic value of circulating calprotectin in the severity of COVID‐19. Eventually, we found that levels of circulating calprotectin were positively associated with the severity and mortality outcome of COVID‐19 patients. Circulating calprotectin has clinical significance and practical value in diagnosing the severity and predicting the prognosis of patients with COVID‐19.

Calprotectin is widely distributed in peripheral blood and fecal. At present, fecal calprotectin has been studied more, which is recognized to be related to intestinal injury.^46^ The cause of gastrointestinal reaction caused by SARS‐CoV‐2 virus may be related to angiotensin‐converting enzyme 2 (ACE2) receptor binding. ACE2 receptors are highly expressed in intestinal cells (e.g., small intestine epithelial cells and large intestine epithelial cells) and are the main route by which viruses enter the intestine.^47^ In addition, severe COVID‐19 patients often have gastrointestinal complications such as ileus, gastrointestinal bleeding, intestinal ischemia, and even intestinal perforation.^48^ Fecal calprotectin is often considered to be an indicator associated with intestinal inflammation, and its most important use is in the differential diagnosis of inflammatory bowel diseases (IBD) and irritable bowel syndrome.^49^ When inflammation occurs in the intestinal tract, fecal calprotectin can be detected in the feces due to the increased permeability of the intestinal mucosa, which allows neutrophils to infiltrate and release calprotectin.^50^ Intestinal injury responds to both acute and chronic inflammatory conditions. Many studies have shown that fecal calprotectin levels are highly expressed in patients with COVID‐19.^51^, ^52^, ^53^ Therefore, SARS‐CoV‐2 virus attacks the ACE2 receptor of gastrointestinal cells after infection, causing gastrointestinal inflammation and complications, which may be the main reason for the increase of calprotectin. But the study had also shown high levels of fecal calprotectin in patients with COVID‐19, regardless of gastrointestinal symptoms.^54^ However, the correlation between the specific mechanism and infection diagnosis is still unclear, which is an important line for future research. Circulating calprotectin has been recognized by some studies as one of the most relevant predictors of COVID‐19 disease severity, and transcriptome analysis has shown that S100A8 and S100A9 are the few genes that are significantly upregulated after infection of SARS‐CoV‐2.^55^, ^56^ From the studies searched, calprotectin is expected to be an accurate and effective biomarker for COVID‐19.

In a meta‐analysis conducted by Udeh et al.,^57^ eight quantitative literatures were included, and the pooled SMD of calprotectin between the cases with severe COVID‐19 and other cases was 1.34 (95% CI: 0.77–1.91). Mao et al.^58^ also included eight quantitative literatures for meta‐analysis, and the pooled SMD was 1.18 (95% CI: 0.74–1.62). The current study included eight articles for meta‐analysis with an effect size of SMD. Compared with the above two studies, the results obtained were more timely, more comprehensive and more persuasive. In the included paper, four methods were applied to detect the scope of calprotectin. Because the applied methods and units were not uniform, we applied SMD to combine them. It can be observed that calprotectin of severe patients is significantly higher than that of mild patients. The normal values of calprotectin measured by different methods can be seen in Table 1. This meta‐analysis showed that the pooled SMD was 1.84 (95% CI: 1.09–2.60). These results suggest that the higher the levels of circulating calprotectin, the more likely patients were severe COVID‐19. In a meta‐analysis of adverse outcomes, OR and its 95% CI were used to assess the significance of circulating calprotectin in predicting mortality in COVID‐19 patients. The results of the meta‐analysis showed that OR and 95% CI were 8.60 (2.17–34.12), which suggested that high expression of circulating calprotectin was a poor prognostic marker for COVID‐19.

In recent years, studies on calprotectin and COVID‐19 have been widely carried out worldwide and were increasing year by year. However, our study was the first meta‐analysis to assess the diagnostic value of calprotectin in the severity of COVID‐19. In this study, the effect results of 11 diagnostic tests in 11 articles were combined, and the pooled sensitivity and specificity of calprotectin in diagnosing the severity of COVID‐19 were 0.75 (95% CI: 0.64–0.84), 0.88 (95% CI: 0.79–0.94), respectively. The PLR for circulating calprotectin on COVID‐19 severity was 6.4 (95% CI: 3.5–11.6). Patients with severe COVID‐19 were 6.4 times more likely to detect positive for circulating calprotectin than patients with mild COVID‐19. The NLR for circulating calprotectin on COVID‐19 severity was 0.28 (95% CI: 0.19–0.42). This indicated that the probability of circulating calprotectin being diagnosed as mild when the gold standard diagnosis was severe was 0.28. The pooled DOR for circulating calprotectin on COVID‐19 severity was 18.44 (95% CI: 9.07–37.51). It suggested that circulating calprotectin had a high diagnostic value for COVID‐19 severity. In this meta‐analysis, circulating calprotectin was moderately effective in the diagnosis of COVID‐19 severity and had outstanding diagnostic value for COVID‐19.

As a potential COVID‐19 biomarker, calprotectin has multiple advantages. First, calprotectin could be used both as a diagnostic tool and as a severity stratification tool for COVID‐19 patients. The levels of circulating calprotectin could discriminate patients who develop a severe form of COVID‐19. Meanwhile, it had an excellent predictive value for adverse outcomes in patients with COVID‐19. Second, the levels of circulating calprotectin correlated better with the severity of COVID‐19 compared with a host of other traditional biomarkers including IL‐6, CRP, ESR, and D‐dimer.^32^, ^34^, ^36^, ^59^ Third, circulating calprotectin offers a clear kinetic advantage as the first indication of acute inflammation since it does not require de novo synthesis.^60^ Moreover, compared with other more common biomarkers, calprotectin had the advantage that marker levels did not increase significantly during ICU due to potential bacterial infection and other influences.^61^ Last but not least, given the encouraging results in treating inflammatory disorders,^62^ S100A8/S100A9 inhibitors (e.g., paquinimod) may be a promising treatment option for severe or critically ill COVID‐19 individuals.^63^

Although there were multiple original studies evaluating the value of calprotectin in COVID‐19 from the aspects of expression level, diagnostic, and prognostic value, their results were controversial. This was the first time to comprehensively assess this issue by using a method of pair‐wise meta‐analysis. By utilizing rigorous statistical methods and multiple outcome estimates, the pooled results were robust and convincing. However, there were limitations existing in the current meta‐analysis. First, all included studies were observational ones and did not specify whether blinding was used or not. Second, after pooling effect sizes, high heterogeneity was found across studies. We guessed that it was caused by different races and various parts and ways of detecting calprotectin. Third, the small number of included patients reduced the strength of the current meta‐analysis. Currently, separate analysis assessing the prognostic value of serum calprotectin regarding intubation is unavailable. Fourth, the mechanism of calprotectin is still in the initial stage. The specific reasons for the elevated expression of calprotectin are unclear as to how it works in viral infections.

## 5 CONCLUSION

In a word, calprotectin was significantly elevated in patients with severe COVID‐19. This atypical cytokine may serve as a novel biomarker for predicting the severity of COVID‐19 with satisfactory diagnostic efficiency. Meanwhile, mortality in patients with COVID‐19 tends to be positively correlated with calprotectin levels. More high‐quality studies are still needed to validate the value of calprotectin as a diagnostic and prognostic marker for COVID‐19, and clarify the function of this heterodimer in disease progression.

## AUTHOR CONTRIBUTIONS

**Haoran Zhang**: Conceptualization; data curation; formal analysis; investigation; methodology; project administration; software; writing—original draft. **Qingyu Zhang**: Conceptualization; data curation; formal analysis; funding acquisition; investigation; methodology; project administration; software; writing—original draft. **Kun Liu**: Formal analysis; software; writing—original draft. **Zenong Yuan**: Writing—review and editing. **Xiqiang Xu**: Conceptualization; investigation; methodology; software; supervision; writing—review and editing. **Jun Dong**: Conceptualization; funding acquisition; project administration; software; supervision; validation; writing—review and editing.

## CONFLICT OF INTEREST STATEMENT

The authors declare no conflict of interest.

## Supporting information

Supplementary Figure 1 Deeks’ funnel plot of calprotectin levels

Supplementary Figure 2 Sensitivity analysis was used to assess whether there was a unstable experiment

Supplementary Figure 3 Deeks’ funnel plot of calprotectin diagnosis value for COVID‐19 severity

## Data Availability

All data generated or analyzed in this study are available in the article.
