# Supplementary figures and images for: Elevated level of circulating calprotectin correlates with severity and high mortality in patients with COVID‐19
Source: Immun Inflamm Dis. 2024 Mar 13;12(3):e1212. doi: 10.1002/iid3.1212 (PMC10936233; doi:10.1002/iid3.1212)

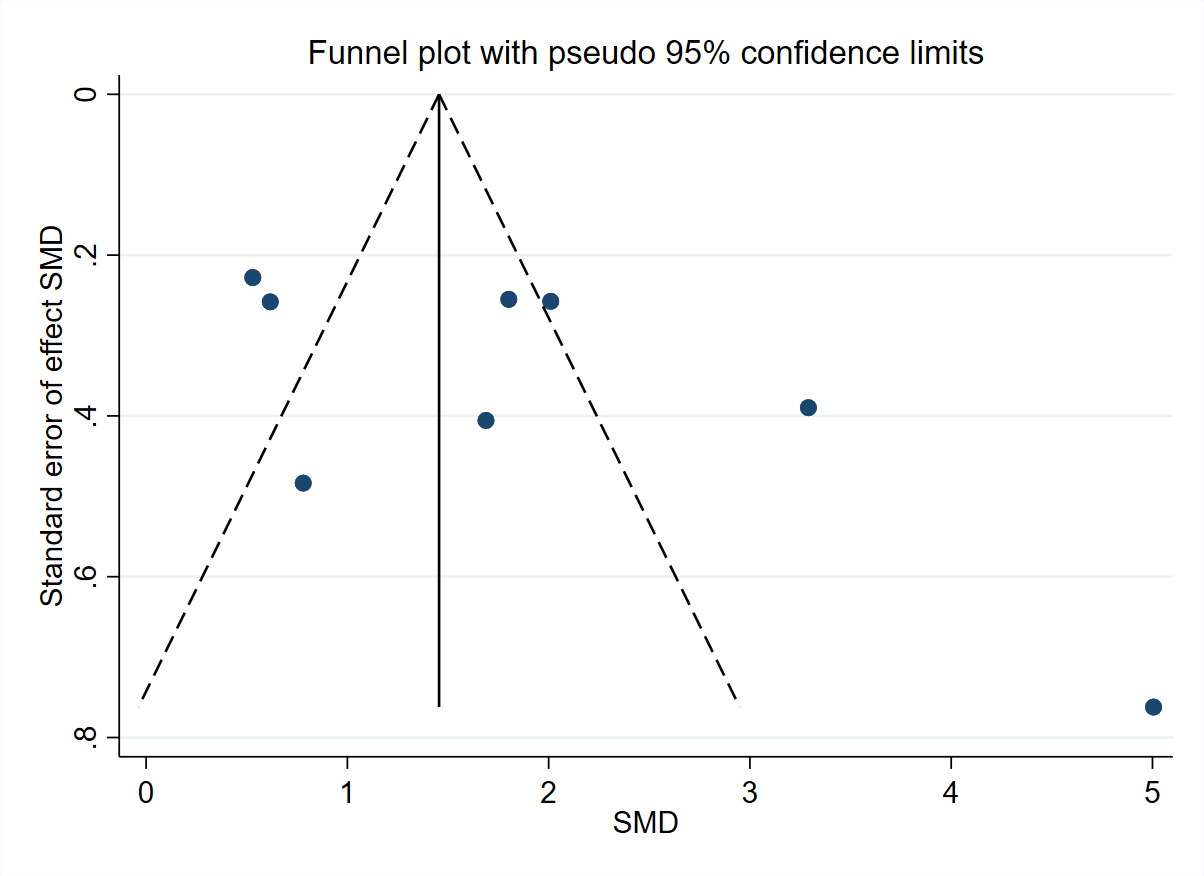

Supplement: Supplementary file 1 — Supplementary Figure 1 Deeks’ funnel plot of calprotectin levels [file IID3-12-e1212-s002.tif]

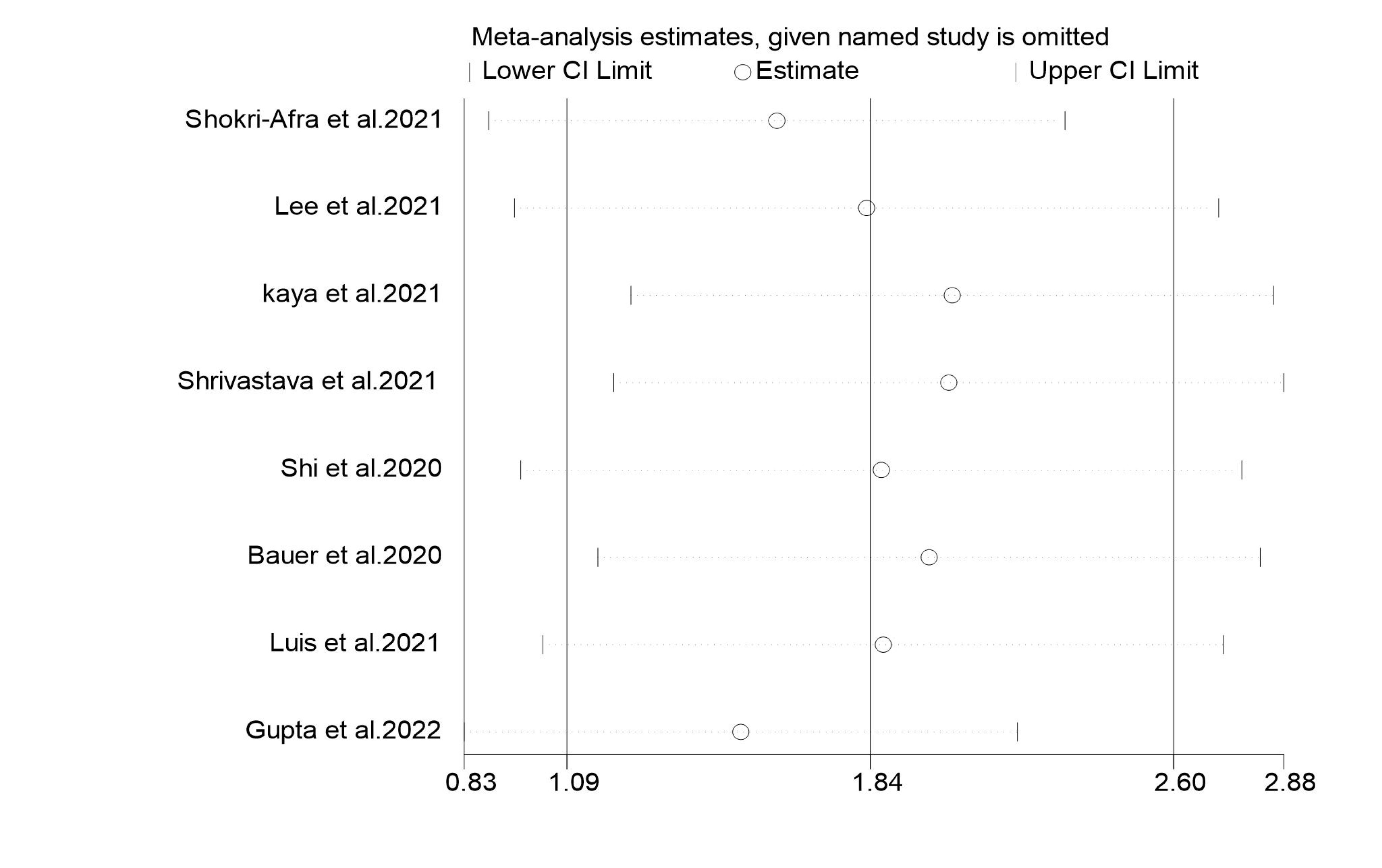

Supplement: Supplementary file 2 — Supplementary Figure 2 Sensitivity analysis was used to assess whether there was a unstable experiment [file IID3-12-e1212-s003.tif]

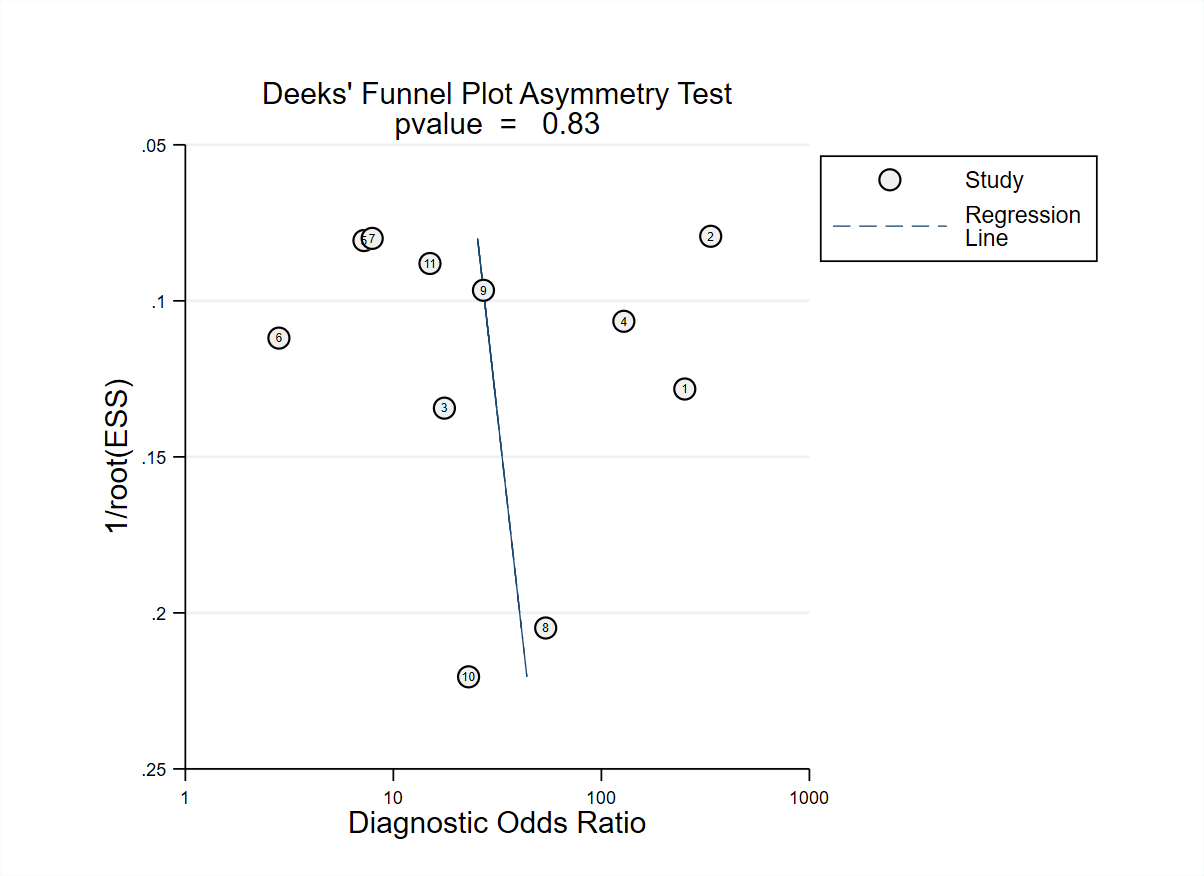

Supplement: Supplementary file 3 — Supplementary Figure 3 Deeks’ funnel plot of calprotectin diagnosis value for COVID‐19 severity [file IID3-12-e1212-s001.tif]
